# Coping strategies of people living with AIDS in face of the disease

**DOI:** 10.1590/1518-8345.2284.2985

**Published:** 2018-03-08

**Authors:** Rafael Tavares Silveira Silva, Richardson Augusto Rosendo da Silva, Iellen Dantas Campos Verdes Rodrigues, Vinicius Lino de Souza, Bárbara Coeli Oliveira da Silva, Francisca Marta de Lima Costa Souza

**Affiliations:** 1 MSc, Coordinator of the undergraduate course in nursing, Faculdade Evolução Alto Oeste Potiguar, Pau dos Ferros, RN, Brazil.; 2 PhD, Adjunct Professor, Departamento de Enfermagem, Universidade Federal do Rio Grande do Norte, Natal, RN, Brazil.; 3 PhD, RN, Serviço de Atendimento Móvel de Urgência, Prefeitura Municipal de Timon, Timon, MA, Brazil. Adjunct Professor, Departamento de Enfermagem, Universidade Federal de Sergipe, Lagarto, SE, Brazil.; Universidade Federal de Sergipe, Departamento de Enfermagem, Universidade Federal de Sergipe, Lagarto, SE, Brazil; 4 MSc, Professor, Departamento de Enfermagem, Universidade Federal do Rio Grande do Norte, Natal, RN, Brazil.; 5 Doctoral Student, Universidade Federal do Rio Grande do Norte, Natal, RN, Brazil.; 6 Doctoral Student, Programa de Pós-Graduação em Enfermagem, Universidade Federal do Rio Grande do Norte, Natal, RN, Brazil. RN, Maternidade Professor Leide Morais, Natal, RN, Brazil. Professor, Departamento de Enfermagem, Universidade Federal do Rio Grande do Norte, Natal, RN, Brazil.; Maternidade Professor Leide Morais, Natal, RN, Brazil

**Keywords:** Adaptation Psychological, Mental Health, Acquired Immunodeficiency Syndrome, HIV, Nursing Care, Psychiatric Nursing

## Abstract

**Objective:**

to identify the coping strategies of people living with aids to face the disease and analyze them according to sociodemographic, clinical and lifestyle variables.

**Method:**

this is a cross-sectional quantitative study. The sample consisted of 331 people living with aids treated at an outpatient clinic at a referral hospital for treatment of aids. The Coping Strategies Inventory was used to collect the data.

**Results:**

emotion-focused coping modes were more frequently mentioned. The mean scores of women, workers, religious people, and people who never withdrew from the treatment were higher for all factors. Patients who had a partner, who lived with family members and who received treatment support, had higher mean scores in coping, withdrawal and social support factors. As for leisure and the practice of physical exercises, the emotion-focused modes also predominated. A correlation was identified between treatment time, schooling, family income and the factors of the Coping Strategies Inventory of.

**Conclusion:**

the study showed that the most frequent coping modes were those focused on emotion.

## Introduction

Acquired Immune Deficiency Syndrome (AIDS) is an emerging disease and one of the major health problems today, due to its pandemic status and severe characteristics. A reduction of mortality rate associated with the success of antiretroviral therapy (ART) has been observed^1^. However, the disease still has a strong impact on the lives of people living with aids, mainly due to stigma and prejudice, psychiatric disorders such as depression, changes in marital relationships such as abandonment of the partner, and difficulties in keeping and reintegrating into the job market with consequent possible financial problems that may negatively influence ART adherence^2^.

Develop coping strategies is necessary to reduce the psychological suffering of people living with aids, arising from all the difficulties related to this diseaseto ^3^. *C*oping strategies consist in thoughts and behaviors that people use as a strategy to organize the internal and external demands of a particular stressing event or factor^4^.

Coping strategies are influenced by factors such as sociodemographic, personal, sociocultural and environmental aspects. They also depend on resources that may be personal, such as health status, morality, religiosity, intelligence and individual peculiarities, but also sociological, such as family characteristics, social networks, economic situation, conjugal relationship, and so forth^5^.

In this sense, the understanding and identification of coping strategies can help nurses and other health professionals to direct interventions to control stressors related to the disease, favoring the adaptive process to the therapeutic regimen. Therefore, studies with this theme are justified by their contribution to improving the quality of life of people living with aids.

Furthermore, the relevance of this research is justified by the need to know better the psychological and social aspects that affect people living with aids so as to structure the care models towards comprehensive and interdisciplinary care, aiming to meet the new psychosocial demands that can emerge in the context of their lives.

In this sense, the present study aimed to identify the coping strategies of people living with aids to face the disease and to analyze them according to sociodemographic, clinical and lifestyle variables.

## Method

This is a cross-sectional study with quantitative approach performed at a referral hospital for the treatment of people living with HIV/aids in Northeast Brazil.

Without-replacement sampling calculation was based on the total number of patients enrolled in the outpatient clinic of the hospital, constituting a population of 2,350 patients. This population was considered the universe for calculation of the sample. The formula for finite populations was used and the criteria adopted were a 95% confidence level (Z∞ = 1.96) and sample error of 5%, resulting in a sample size of 331 patients.

The adopted selection criteria were: having been clinically diagnosed with aids; age over 18 years; having used antiretroviral therapy for at least 6 months; being registered at the outpatient clinic of the hospital during the data collection period. We excluded people with neurological deficits, pregnant women, distressed people and who were undergoing post-exposure prophylaxis. Recruitment took place by convenience and patients that met the inclusion criteria were actively searched. The search took place in a consecutive manner, that is, patients were invited after outpatient consultations to participate in the study. All the ethical aspects inherent to research involving human beings were respected. Data were collected through interviews conducted from February to August 2015.

To evaluate the coping strategies related to aids and treatment, the study population responded to the Coping Strategies Inventory (CSI)^6^, a checklist with Likert-type scales. The CSI has been translated and validated into the Portuguese language^6^ ^)^ and has confirmed correspondence with the original English version, allowing its use in other studies.

The inventory consists of 8 coping factors, classified as follows: problem-focused coping (factors: confrontation and problem solving), emotion-focused coping (factors: withdrawal, self-care, responsibility acceptance, positive reappraisal and escape-avoidance) and problem- and emotion-focused coping (factor: social support)^6^.

The analysis was based on descriptive and inferential statistics. For this, the data obtained in the CSI were organized in a spreadsheet in the *Excel for Windows* 2010. The spreadsheet was then imported into the Statistical Package for Social Sciences (SPSS) version 20.0 to analyze univariate frequencies, contingency tables, measures of centrality (mean and median) and of dispersion, as standard deviations (SD), and to calculate the Cronbach’s alpha coefficient.

A t-test and the Mann-Whitney test were used to compare the mean scores of the CSI factors and of sociodemographic, clinical and lifestyle variables, for we had two independent samples. The Spearman correlation test was used to analyze continuous variables such as treatment time, schooling and income, considering r values of 0.10 to 0.39 (weak); r values of 0.40 to 0.69 (moderate); and r values of 0.70 to 1.0 (strong). Comparison and correlation scores at p < 0.006 were considered statistically significant according to the Bonferroni criterion, as p = 0.05 was divided by the eight CSI factors.

The research was evaluated by the Research Ethics Committee of the responsible institution and was granted approval with Certificate of Presentation for Ethical Appreciation (CPEA) nº 16578613.0.0000.5537. All participants of the study provided written consent when invited to participate. 

## Results

A total of 331 people participated in the study, of which 172 (52%) were males and 159 (48%) were females. Age ranged from 20 to 63 years, with an average of 41.5 years and a standard deviation (SD) of 13.5 years. Regarding schooling, the mean number of years was 7.5 years (SD = 4.5 years). Regarding the marital situation, 132 (40%) had a partner and 132 (40%) lived with relatives. When asked if someone followed their treatment, 94 (28%) people gave affirmative answers.

The monthly family income reported varied from less than 01 to 05 minimum wages (MW), with a mean of 1.8 MW (SD = 1.2 MW); the predominant income range was 01 MW, in the case of 225 (68%) people. In relation to work, 265 people (80%) reported having some work-related paid activity. Table 1 presents the mean scores, standard deviation, median, and Cronbach’s alpha (α) coefficient of each factor.

**Table 1:** Mean scores of the Coping Strategies Inventory factors applied to people living with AIDS. Natal, RN, Brazil, 2015

Factors (number of items)	Mean score	Standard deviation	Median	(Cronbach’ alpha)
Confrontation (6)*	0.80	0.66	0.70	0.84
Withdrawal (7)^†^	1.06	0.70	1.06	0.86
Self-control (5)^‡^	1.27	0.61	1.27	0.80
Social support (6)^*^	1.31	0.69	1.24	0.86
Responsibility acceptance(7)^†^	1.14	0.74	1.06	0.81
Escape-avoidance (2)^§^	1.78	0.93	1.67	0.82
Problem solving (4)^||^	1.80	0.96	1.74	0.91
Positive reappraisal (9)^¶^	1.86	0.84	1.78	0.95

* (6) - 6 items, †(7) - 7 items, ‡(5) - 5 items, §(2) - 2 items, ||(4) - 4 itens, ¶(9) - 9 items.

The internal consistency of the CSI factors, measured by the Cronbach’s alpha coefficient, ranged from 0.80 (in the self-control factor) to 0.95 (in the positive reappraisal factor). In this study, there was a greater reference to the coping modes related to the positive reappraisal factor, with a mean score of 1.86, and a lower reference to the coping modes related to the confrontation factor, with a mean score of 0.80. There was a predominance of emotion-focused coping modes. The scores of comparison of the means of the CSI factors according to sociodemographic variables are presented in Table 2.

**Table 2:** Comparison scores of the means of the factors of Coping Strategies Inventory applied to people living with AIDS, according to sociodemographic variables. Natal, RN, Brazil, 2015

Sociodemographic variables	Comparison scores of the means of the Coping Strategies Inventory factors							
	Confrontation	withdrawal	Self-control	Social support	Responsibility acceptance	Escape-avoidance	Problem solving	Positive reappraisal
Gender								
Male	0.70	1.58	1.82	2.09	1.74	2.13	2.12	2.23
Female	0.80*	1.67*	1.94*	2.13	1.80*	2.27*	2.33	2.42*
Age group								
18-59	0.75	1.55	1.94	2.13	1.80	2.14	2.33	2.42
> 60	0.86	1.62*	1.81*	2.07	1.72	2.27	2.21	2.22*
Paid work								
Yes	0.89*	1.62*	1.94*	2.13	1.80*	2.27*	2.33	2.42*
No	0.73	1.54	1.83	2.07	1.70	2.18	2.23	2.31
Partner								
Yes	0.83*	1.62*	1.88	2.13*	1.70	2.24	2.27	2.35
No	0.72	1.52	1.92	2.05	1.78	2.26	2.31	2.41
People in the household								
Lives alone	0.71	1.52	1.84	2.09	1.74	2.17	2.20	2.42
With family	0.80*	1.60*	1.93*	2.12	1.80*	2.25*	2.33*	2.37
Treatment support								
Yes	0.86	1.62*	1.94	2.13	1.80*	2.27	2.33*	2.42*
No	0.74	1.54	1.85	2.07	1.72	2.20	2.21	2.34
Religion								
Yes	0.87	1.62*	1.94*	2.13	1.80	2.27	2.33	2.42*
No	0.73	1.52	1.85	2.10	1.74	2.12	2.11	2.28

* Statistically significant scores at p < 0.006.

The mean scores of women were higher than those of men in all factors and were statistically significant for the factors: confrontation, withdrawal, self-control, responsibility acceptance, escape-avoidance and positive reappraisal.

Elderly people (60 years of age or older) presented higher mean scores on the confrontation, withdrawal and escape-avoidance factors, with predominantly emotion-focused coping modes. Withdrawal, self-control and positive reappraisal were statistically significant.

Concerning occupation, the average scores of people who worked were higher than those who did not work in all factors, being statistically significant for confrontation, withdrawal, self-control, responsibility acceptance, escape-avoidance, and positive reappraisal.

People who reported having a partner had higher mean scores in the confrontation, withdrawal and social support factors than the people who mentioned having no partner. This, the problem-focused and emotion-focused coping modes were similarly presented. However, only the confrontation, withdrawal and social support factors were statistically significant.

People who reported living with family members had higher mean scores in almost all factors, except for the positive reappraisal factor, than those who reported living alone. Therefore, emotion-focused and problem-focused modes were presented. The following factors were statistically significant: confrontation, withdrawal, self-control, responsibility acceptance, escape-avoidance, and problem solving.

People who reported having some treatment support had higher mean scores in all factors than people who did otherwise. There was statistical significance for the factors of withdrawal, responsibility acceptance, problem solving and positive reappraisal.

People who practiced some religion also had higher average scores in all factors compared to non-practitioners. The statistically significant coping modes were: withdrawal, self-control, and positive reappraisal.

The comparison between mean scores of the CSI factors according to sociodemographic variables showed the predominance of the emotion-focused coping modes. However, the confrontation factor, a coping mode focused on the problem, stood up in all the variables used, besides presenting statistical significance in five of the eight variables. 

Comparison scores of the means of the CSI factors according to clinical variables are presented in Table 3 and also show a predominance of emotion-focused modes, and the confronting factor appears in all the variables used.

**Table 3:** Comparison scores of the means of the Coping Strategies Inventory factors applied to people living with aids according to clinical variables. Natal, RN, Brazil, 2015

Clinical variables	Comparison scores of the means of the Coping Strategies Inventory factors							
	Confrontation	withdrawal	self-control	Social support	Responsibility acceptance	Escape-avoidance	Problem solving	Positive reappraisal
Comorbidity								
Yes	0.71	1.51	1.86	2.09	1.74	2.26	2.28	2.32
No	0.89	1.60	1.90	2.02	1.79	2.20	2.12	2.40
CD4								
< 200 cell/mm^3^	0.71	1.60	1.94	2.04	1.75	2.27	2.30	2.40
> 200 cell/mm^3^	0.89	1.56	1.83	2.13	1.80	2.07	2.28	2.31
Treatment abandoned								
Yes	0.72	1.56	1.84	2.08	1.72	2.18	2.21	2.31
No	0.88	1.62	1.94	2.13*	1.80*	2.27*	2.33	2.42

* Statistically significant scores at p < 0.006.

The highest mean scores related to people without comorbidities were confrontation, withdrawal, self-control, responsibility acceptance and positive reappraisal, with a predominance of emotion-focused modes. However, none of the factors presented statistical significance.

As for CD4 cell counts, higher mean scores were observed for CD4 cell counts > 200 cell/mm^3^ only in the confrontation, social support and responsibility acceptance factors; no one factor was statistically significant. In this respect, therefore, the three coping modes were equally observed.

People who never abandoned treatment also had higher mean scores in all factors than those who had done so. Statistical significance was only observed for social support, problem acceptance, and escape-avoidance. Table 4 presents the comparison scores of the means of the CSI factors in relation to life habits of the study population.

**Table 4:** Comparison scores of the Coping Strategies Inventory factors applied to people living with aids according to life habits. Natal, RN, Brazil, 2015

Lifestyle variables	Comparison scores of the means of the Coping Strategies Inventory factors							
	Confrontation	Withdrawal	Self-control	Social support	Responsibility acceptance	Escape-avoidance	Problem solving	Positive reappraisal
Recreation								
Yes	0.84	1.62	1.86*	2.03*	1.68*	2.26*	2.26*	2.40*
No	0.76	1.54	1.90	2.11	1.79	2.19	2.32	2.47
Practice of physical activity								
Yes	0.88	1.61	1.92*	2.11*	1.78*	2.26*	2.33*	2.38*
No	0.72	1.57	1.81	2.04	1.71	2.18	2.25	2.41

* Statistically significant scores at p < 0.006.

Regarding leisure and physical exercise, it was observed that the mean scores were higher for confrontation, withdrawal, and escape-avoidance in individuals who have leisure, being statistically significant for the withdrawal and escape-avoidance modes. Individuals who practiced some physical activity had higher scores in almost all factors, except for positive reappraisal; significant results were found for the withdrawal and self-control modes. Therefore, in both groups, emotion-focused modes predominated as happened in all the other variables used. 

In the analysis of continuous variables related to the CSI factors found statistical significance, in which the Spearman correlation test was applied, showing statistically significant correlations (Table 5).

**Table 5:** Correlation between the Coping Strategies Inventory factors and the time of treatment, schooling and monthly family income in people living with aids.

Spearman correlation Coping Strategies Inventory factors																
Variables	Confrontation		Withdrawal		Self-control		Social support		Responsibility acceptance		Escape-avoidance		Problem solving		Positive reappraisal	
	r*	p^†^	r*	p^†^	r*	p^†^	r*	p^†^	r*	p^†^	r*	p^†^	r*	p^†^	r*	p^†^
Treatment time in months	0.239	0.005^‡^	0.126	0.005^‡^	0.213	0.052	0.024	0.044	0.275	0.001^‡^	0.342	0.005^‡^	0.262	0.035	0.122	0.094
Schooling (years)	0.275	0.003^‡^	0.225	0.002^‡^	0.263	0.623	0.115	0.065	0.268	0.003^‡^	0.286	0.003^‡^	0.431	0.068	0.068	0.163
Monthly family income (MW)	0.315	0.004^‡^	0.284	0.004^‡^	0.245	0.008	0.305	0.071	0.342	0.004^‡^	0.362	0.001^‡^	0.487	0.023	0.142	0.254

* r - correlation value, †p - p value, ‡Statistically significant scores for p < 0.006.

In the variables treatment time, schooling, and family income, Spearman correlation indices were significant for comfort, withdrawal, responsibility acceptance, and escape-avoidance, thus predominantly focused on emotion, although the intensity was weak in almost all variables.

## Discussion

People living with AIDS face several stressors related to the chronicity of the disease and/or the ART. In this sense, the application of the CSI^6^ was useful to identify how these people face such condition in their daily life.

Coping arises from the idea of overcoming stressors, and in the present study, we identified higher means related to the positive reappraisal, problem solving and escape-avoidance factors, demonstrating the more frequent use of problem-focused and emotion-focused coping methods.

A study of people living with aids identified the use of coping methods focused on positive reappraisal and escape-avoidance factors, thus focused on emotion. The main coping strategies used were maintaining confidentiality about their seropositive condition, optimism towards treatment, search for social support, rationalization, social comparison, spirituality/religiosity, avoidance and distraction^7^.

In this sense, they used positive reappraisal in order to create new meanings for the stress, facing it with a positive attitude, leading to personal growth. Thus, they attribute new meanings to the disease in order to see it from a different perspective, that is, a positive perspective^8^. Escape-avoidance is a way people use to escape problems. There are denial and lack of interest in dealing with disease-related stressors, which may negatively contribute for improving the clinical picture, as it may seem like a way of fantasizing possible solutions for the problems, without actually taking concrete actions to modify the reality^9^.

A study identified that positive coping through avoidance and social isolation has a greater association with mental health problems such as depression^10^. Furthermore, passive coping represents a solution to patients who believe that there are no measures capable of counteracting the stressors associated with the disease, leading to other disorders such as propensity to alcohol abuse in order to escape from the problem^11^.

The occurrence of higher mean scores among women in all factors may be related to the fact that they are often abandoned by their partners after a positive HIV diagnosis. Then, alone in the face of the infection, they have to combat the effects caused by the disease and somehow move forward, taking care of their children, who dependend on them^12^.

Another important aspect of coping with the disease is the job because it can give meaning to people’s lives. One study demonstrated that job constitutes a form of support to positively face the condition of living with AIDS, as it promotes confidence and self-esteem^12^. In line with this, the present study identified higher mean scores in all factors, as well as statistical significance for almost all factors, among individual who worked, suggesting that they in some way seek to overcome the problems arising from the disease.

Having a partner, living with someone and having support during the treatment led to higher mean scores in several factors. However, they coincided in the modes focused on confrontation, withdrawal and social support. This suggests that the affective family context can influence the way people living with aids face the disease, i.e. in a positive way. The confrontation factor demonstrates efforts to change the situation and the social support factor describes efforts to obtain information and support aiming at overcoming^13^.

A study showed that among the main psychosocial challenges this group of people has to face are discrimination, stigma, and challenges of establishing loving relationships if their seropositive condition is known. Fear of having their diagnosis revealed can lead them to isolate themselves from family and friends, which intensifies emotional distress and consequently reflecting on their ability to adhere to drug therapy and seek social support. Among the strategies used to deal with this situation, social support, confidentiality regarding the diagnosis, optimism, rationalization, social comparison and religiosity/spirituality^8^ ^)^ were mentioned by the participants.

People who had a religion had higher mean scores in all factors, and thus they used both emotion-focused and problem-focused coping strategies. This fact is possibly associated with the idea that religiosity can be a coping strategy^14^. Religion and spirituality among people living with aids are used as strategies to deal with the adversities arising from the disease^15^.

Regarding the clinical variables, the most statistically significant was the non-abandonment of treatment. Mean scores were higher in all factors among people who never quit the treatment compared to those who had already abandoned it. The factors that were statistically significant such as social support and responsibility acceptance reinforced the idea of ​​adherence as a result of positive coping. However, the escape-avoidance factor also presented statistical significance and may be related to the difficulties encountered during treatment such as the large number of pills to take, rigid schedules and routines, side effects, long treatment time, and so forth^16^ ^-^ ^17^.

When it comes to life habits, with the exception of the confrontation and withdrawal factors, all other variables presented statistical significance. This suggests that leisure and practice of physical activity may be associated with a way of avoiding the problems arising from the disease. Physical exercise and leisure were also pointed out in a study as strategies of escape and relief from stress, depression and low self-esteem triggered by the condition of living with aids^10^.

In the present study, Spearman correlation coefficients between the continuous variables (treatment time, schooling and family income) and the CSI factors showed statistical significance for the factors focused on the emotion (withdrawal, responsibility acceptance, and escape-avoidance ), although with weak intensity.

Emotion-Focusing coping can be seen as positive action because when the attitudes of the person living with aids aim to solve the problem arising from the disease, there is a positive confrontation. This happens when confrontation is focused on accepting responsibility since this factor is characterized by attitudes of contribution of the person in the search for knowledge about the disease and by trying to do the right thing^9^. In this case, optimism is positively associated with psychological well-being and the reduction of stigma associated with aids^18^.

As contributions to the advancement of scientific knowledge, we emphasize that the identification of the coping modes used by people living with aids is fundamental for the nursing and the health teams in the perspective of setting goals and planning appropriate interventions to the real needs of this population who suffer so much from changes in daily life imposed by disease, stigma, prejudice, and discrimination.

The limits of the study were related to the fact that the research was carried out with a specific population, people living with aids. Thus, coping studies should be encouraged for other populations, considering that research on this theme may subsidize actions that allow the development of the adaptive mechanisms of people living with chronic diseases and their families.

## Conclusion

The study identified higher means for emotion-centered coping methods used by people living with aids, that is, methods related to the positive reappraisal factor.

Women and people who work possibly used emotion-focused and problem- focused coping strategies. The family affective context and religiosity can influence the confrontation of the disease in a positive way. Adherence to treatment was identified as having a positive effect on coping. Leisure and physical activity may be associated with a way to escape the problems brought about by the disease. As for the time of schooling and the family income, we believe that education and the financial resources influence the ways of facing the disease.
